# Light‐Induced Pulsed EPR Dipolar Spectroscopy on a Paradigmatic Hemeprotein

**DOI:** 10.1002/cphc.201900139

**Published:** 2019-03-21

**Authors:** Maria Giulia Dal Farra, Sabine Richert, Caterina Martin, Charles Larminie, Marina Gobbo, Elisabetta Bergantino, Christiane R. Timmel, Alice M. Bowen, Marilena Di Valentin

**Affiliations:** ^1^ Department of Chemical Sciences University of Padova Via Marzolo 1 35131 Padova Italy; ^2^ Centre for Advanced Electron Spin Resonance (CAESR) Department of Chemistry, Inorganic Chemistry Laboratory University of Oxford South Parks Road Oxford OX1 3QR UK; ^3^ Department of Biology University of Padova viale G. Colombo 3 35121 Padova Italy; ^4^ *current affiliation*: Institute of Physical Chemistry University of Freiburg Albertstr. 21 79104 Freiburg Germany; ^5^ *current affiliation*: Groningen Biomolecular Science and Biotechnology Institute University of Groningen 9700 AB Groningen The Netherlands

**Keywords:** DEER/PELDOR, EPR spectroscopy, heme proteins, porphyrinoids, triplet state

## Abstract

Light‐induced pulsed EPR dipolar spectroscopic methods allow the determination of nanometer distances between paramagnetic sites. Here we employ orthogonal spin labels, a chromophore triplet state and a stable radical, to carry out distance measurements in singly nitroxide‐labeled human neuroglobin. We demonstrate that Zn‐substitution of neuroglobin, to populate the Zn(II) protoporphyrin IX triplet state, makes it possible to perform light‐induced pulsed dipolar experiments on hemeproteins, extending the use of light‐induced dipolar spectroscopy to this large class of metalloproteins. The versatility of the method is ensured by the employment of different techniques: relaxation‐induced dipolar modulation enhancement (RIDME) is applied for the first time to the photoexcited triplet state. In addition, an alternative pulse scheme for laser‐induced magnetic dipole (LaserIMD) spectroscopy, based on the refocused‐echo detection sequence, is proposed for accurate zero‐time determination and reliable distance analysis.

Electron paramagnetic resonance (EPR) pulsed dipolar spectroscopy (PDS) is an important biophysical technique for studying complex biological assemblies.^1^, ^2^, ^3^ PDS groups a series of pulse EPR techniques that allow the measurement, via the dipolar electron‐electron coupling between two paramagnetic species, of distances and distance distributions. Structural information in the range between 1.6 and 8 nm is obtained with high precision and reliability, while the limit of 16 nm is reached under full deuteration of the sample and solvent.^4^, ^5^, ^6^ Among the PDS techniques, double electron‐electron resonance (DEER), also known as pulsed electron double resonance (PELDOR), is the most frequently used due to its robustness.^7^, ^8^ Other EPR techniques for measuring electron‐electron dipolar couplings include Double‐Quantum Coherence (DQC)^9^ and relaxation‐induced dipolar modulation enhancement (RIDME).^10^, ^11^

Conventionally, PDS measurements are performed between two nitroxide spin labels, attached to proteins by site‐directed spin labelling (SDSL) of a cysteine residue or of a non‐native amino acid, which has been genetically encoded.^12^, ^13^, ^14^ The most commonly used spin label is (1‐oxyl‐2,2,5,5‐tetra‐methylpyrroline‐3‐methyl)‐ methanethiosulfonate (MTSSL), which specifically reacts with the thiol group of cysteine residues.^15^ Triarylmethyl (trityl) radicals are emerging as carbon‐centered spin labels with interesting spectroscopic properties^16^, ^17^, ^18^ while, among metal‐based tags, Gd(III) has proven to be an attractive alternative to radicals for PDS applications at high field.^19^ Recently, Cu(II) and high spin Mn(II) tags have also been successfully employed.^20^, ^21^, ^22^, ^23^

The search for alternative spin labels is an active area of research.^24^ One important new development is the demonstration that the triplet state of porphyrin chromophores can be exploited to determine inter‐spin distances. The first work in this area was conducted on a peptide‐based molecular ruler containing a nitroxide probe and porphyrin moiety.^25^, ^26^ The large electron spin polarization of the photoexcited triplet state,^27^, ^28^ and the consequently high sensitivity of the experiment, furthermore allowed light‐induced PDS methodology to be applied to a photosynthetic protein, containing an endogenous carotenoid triplet state probe.^29^ The dipolar measurements were performed with light‐induced DEER (LiDEER),^25^, ^26^ a variation of the conventional 4‐pulse DEER sequence where a laser pulse is used to generate the triplet state before the application of dichromatic microwave pulses: the detection frequency is resonant with the photo‐induced porphyrin triplet and the pump is resonant with the stable nitroxide radical. In the meantime, a new technique: laser‐induced magnetic dipole (LaserIMD) spectroscopy, based on optical switching of the dipole‐dipole coupling, was proposed as an alternative for triplet‐nitroxide dipolar spectroscopy on the same porphyrin‐based model system.^30^ Comparison between the two techniques was carried out both at X‐band and at Q‐band.^31^, ^32^, ^33^ It was found that the relative signal‐to‐noise of the two techniques depends strongly on the degree of excitation that can be achieved by the pump pulse used in LiDEER, the laser excitation, the relative relaxation times of the two species being investigated and the inter spin distance range that needs to be probed. LaserIMD and LiDEER can therefore be seen as complementary to one another.

Intrinsic paramagnetic centers in biomolecules are ideal spin probes for PDS applications. They are usually fixed rigidly within their parent biomolecule resulting in very accurate and narrow inter‐spin distance distributions. In parallel, combining the nitroxide and the endogenous probe in an orthogonal labelling approach has proven to be very effective since the spectroscopically non‐identical labels can be addressed selectively during the PDS experiment.^34^ Traditionally the research of native paramagnetic probes has been focused on metal‐based centers involving Cu(II), low‐spin Fe(III), iron sulfur and manganese clusters.^35^, ^36^, ^37^, ^38^ Recently, it has been shown that the RIDME experiment is better suited than DEER for distance measurements between spin active moieties with different spin‐lattice relaxation times or species with very broad spectra such as metal ions like low‐spin Fe(III).^39^ Many biological macromolecules, photosynthetic proteins *in primis*, and also proteins belonging to other classes, like hemeproteins and flavins, contain a photoactive cofactor, which, in principle, can be exploited as an endogenous paramagnetic center. Tentatively, the hemeprotein cytochrome C, spin labelled with MTSSL at the free cysteine position, was investigated in order to demonstrate that LaserIMD could be employed for distance measurements between the endogenous prosthetic group and a nitroxide label.^30^ However, no triplet state was observed by EPR spectroscopy, as expected for a low‐spin ferric heme.

In this work, human neuroglobin was chosen as a benchmark hemeprotein to demonstrate the feasibility of the dipolar spectroscopy experiment between a triplet state, photo‐generated on the porphyrin‐derivative group, and a nitroxide probe attached to one of the native cysteines of the protein via SDSL. Human neuroglobin is a good model system in this respect because both a high resolution X‐ray structure^40^ and DEER data^41^, ^42^ are available. On the same protein, M. Ezhevskaya *et al*.^41^ reported DEER measurements exploiting the low‐spin Fe(III) ion of the heme group as an endogenous probe. Here, we replaced the heme cofactor with the Zn(II) protoporphyrin IX (ZnPP)^43^ in order to introduce a photo‐generated triplet state spin label. Following the nomenclature by M. Ezhevskaya *et al*. the mutant G19 of neuroglobin has been prepared (see the Supporting Information for details). The mutant after substitution of the heme cofactor and SDSL with the MTSSL probe is referred to as ZnG19 (see Figure 1).

**Figure 1:**
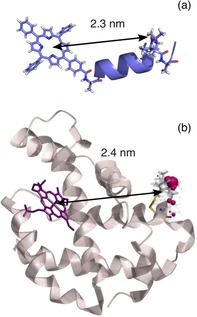
(a) Structure of the model peptide based on DFT data.^20^ The distance between the center of the tetraphenylporphyrin and the N−O midpoint is indicated. (b) Structure of human neuroglobin (PDB: 4MPM).^40^ The distance between the center of the ZnPP and the average position of the MTSSL rotamers computed with the software MMM (Multiscale Modelling of Macromolecules),^44^ is indicated. Details are reported in the Supporting Information.

In parallel, an alternative pulse scheme for LaserIMD, based on the refocused‐echo detection sequence (ReLaserIMD), is proposed in this work in order to ensure accurate zero‐time determination and a more reliable distance analysis. The versatility of the light‐induced dipolar methodology is proven by extending its applicability to this important class of proteins and employing different PDS techniques. In addition to LiDEER and the novel 4PLaserIMD variant, light‐induced dipolar modulation enhancement (LiRIDME) is applied for the first time.

Optimization of the pulse sequences is crucial to broaden the scope of light‐induced PDS. For this purpose we employed an α‐helix peptide, used in previous studies,^25^, ^26^, ^30^ labeled with a tetra‐phenylporphyrin moiety and with the unnatural amino acid TOAC (4‐amino‐1‐oxyl‐2,2,6,6‐tetra‐methylpiperidine‐4‐carboxylic acid). The chemical structure of the model peptide is shown in Figure 1.

In order to analyze the dipolar oscillations accurately and relate this information to an inter‐spin distance distribution, it is fundamental to pinpoint the zero‐time of the experiment precisely. The correct determination of the zero‐time is particularly important for short inter‐spin distances which give rise to high frequency dipolar oscillations. The absence of symmetry in the complete LaserIMD trace (see the Supporting Information) does not allow the symmetry‐based procedure for zero determination to be used in the analysis our experimental data as proposed by Hintze *et al*.^30^ For this reason, in a technique we dub ReLaserIMD (Figure 2 right), we employ the same principle as in the 4‐pulse DEER scheme, in which a refocused echo detection sequence is utilized, to yield a symmetric zero‐time.^8^ The performance of LaserIMD and ReLaserIMD for the model peptide (Figure 1(a)) is compared in Figure 2.

**Figure 2:**
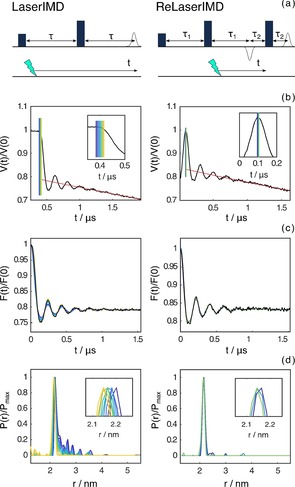
Influence of the zero‐time determination on the distance analysis in LaserIMD and ReLaserIMD measured on the model peptide. For both datasets: pulse sequences (a), raw dipolar time traces with the selected zero time positions used in the distance analysis (b), form factors with the corresponding fits (c) and distance distributions (d) obtained by DeerAnalysis.^45^ The experimental conditions and the parameters of the data analysis are reported in the Supporting Information.

In order to demonstrate convincingly that the ReLaserIMD sequence allows an accurate determination of the zero‐time, the distance analysis of both experimental traces was performed repeating the Tikhonov regularization procedure, implemented in DeerAnalysis,^45^ for a selected set of zero times. In the LaserIMD trace several points could be picked as potential zero times in the zone where the change of slope between the baseline and the drop of the first modulation occurs, preventing the procedure being free from bias. Instead, in ReLaserIMD they can be reasonably restricted to a much smaller range at the top of the first modulation based on the symmetry of the first modulation. This important parameter affects the output of the distance analysis: the different distributions obtained from LaserIMD have their maxima spread over a range of distances of about 0.1 nm, whereas this interval is limited to about 0.01 nm for ReLaserIMD. Furthermore, spurious peaks appear in the distance distribution plot in the case of the standard LaserIMD experiment. For the LaserIMD data set, the result which gave the closest agreement to the ReLaserIMD result was obtained by selecting a zero time in a region where the drop of the first modulation has already started (yellow lines in left panels of Figure 2). This indicates that the experimental zero‐time does not occur when the light flash coincides with the start of the first microwave pulse but rather at some time after this, the exact value of which will depend on the length of the microwave pulse and laser pulse. Thus while the LaserIMD experiment is free from experimental dead‐time due to pulse overlap,^30^ there is still a shift in the zero time, which could be considered a zero‐time artefact, arising from the finite length of the pulses.

Next, the ReLaserIMD sequence was employed, together with LiDEER, to study the dipolar interaction between the triplet state of ZnPP and the nitroxide radical in ZnG19 and prove the feasibility of the light‐induced PDS experiment on heme proteins. Additionally, for the first time, the LiRIDME sequence, in the five‐pulse dead‐time free version, is applied to a triplet probe providing evidence that the longitudinal relaxation properties of the triplet state can be favorable for the application of this technique. The pulse sequences are reported in Figure 3 alongside the corresponding experimental time traces and distance distributions. The ReLaserIMD data set is good, characterized by a modulation depth of 18 % and a signal to noise S/N ≃ 49. This allows more than two well‐resolved periods of the dipolar modulation to be observed, as seen in Figure 3 violet trace. By comparison the LiDEER experiment gives a very poor result, with a high level of noise and a low modulation depth (see the Supporting Information). Each of the two methods has its own specific factors influencing the value of the modulation depth as previously discussed: it depends on the excitation efficiency of the pump pulse for LiDEER and on the laser excitation and quantum yield for (Re)LaserIMD.^31^, ^32^

**Figure 3:**
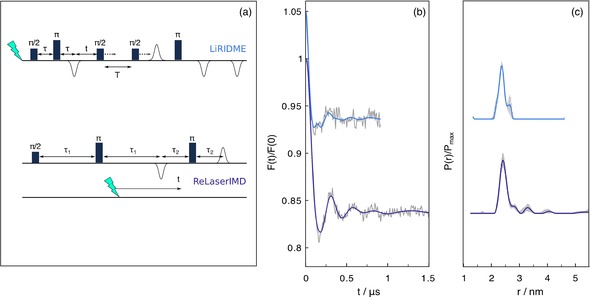
PDS data measured on ZnG19: (a) LiRIDME and ReLaserIMD pulse schemes, (b) form factors (grey) and best fits to the LiRIDME (azure) and the ReLaserIMD (violet) data and (c) corresponding distances distributions. The distance analyses have been performed with DeerAnalysis, for ReLaserIMD, and with OvertoneAnalysis for LiRIDME (with 50 % contribution of the second harmonic overtone). The error bars have been obtained using the validation procedure, implemented in both softwares, varying the starting point for the background fitting between 300 and 500 ns and adding 50 % of the original noise. The experimental conditions are reported in the Supporting Information.

RIDME has previously been shown to be more sensitive than DEER for measuring inter‐spin interactions between paramagnetic species with different longitudinal (T_1_) relaxation times and in the presence of broad EPR spectra.^39^ To this end, LiRIDME (see Figure 3 for pulse sequence) detecting on the nitroxide signal and allowing the broad triplet species to relax was also measured. This set up was favourable as the nitroxide T_1_ is longer than the triplet state relaxation/lifetime. The relaxation and kinetics behaviour (at 20 K as for the PDS experiment) was characterized in detail and it is reported in the Supporting Information. The LiRIDME time trace features a modulation depth of 11 % and a S/N≃18, azure trace Figure 3. The presence of the overtones in the data set, seen as a faster oscillation, particularly evident in the first modulation period, originate from Δm_s_>1 transitions of the triplet state and have been considered in the analysis of distance distributions.^46^

Distance analysis, together with the validation procedure, was performed for all data sets recorded on the ZnG19 protein using DeerAnalysis^45^ or OvertoneAnalysis^46^ in the case of the LiRIDME datasets, and the same most‐probable distance (2.4 nm) and similar distance distributions were obtained in all cases. The excellent agreement of the experimental results with the distance predicted by MMM^44^ analysis based on the X‐ray structural data on the protein (see Figure 1) demonstrates that the triplet state, photo‐generated on the prosthetic group after the ZnPP‐substitution protocol, can be successfully exploited to determine accurate inter‐spin distances in heme‐proteins.

Moreover, the availability of diverse pulse schemes that can be applied in systems containing photoexcited triplet states allows one to select, case‐by‐case, the technique that warrants the best performance in term of S/N. The performance of the three different PDS sequences can be rationalized in terms of the relaxation behavior of the triplet state and the nitroxide probes.

The relative phase memory times of the stable radical and triplet state make either LiDEER or (Re)LaserIMD the most suited experiment in terms of S/N. While LiDEER uses the triplet signal for detection and thus depends on the transverse relaxation time of the triplet, (Re)LaserIMD, using the stable radical for observation, is influenced by the phase memory time of this species. This is the reason why, in the specific case of neuroglobin, where the phase memory time of ZnPP triplet state is of the order of 500 ns only, the use of the LiDEER is almost precluded, despite the favorable spin polarization of the triplet (see Figure S2 in the Supporting Information).^47^

LiRIDME works favorably when the two species under investigation have different longitudinal relaxation times and the slower relaxing species is used for detection.^39^ The longitudinal relaxation time/lifetime of the ZnPP triplet state is faster than that of the nitroxide, similar to the relationship found between metal centers and nitroxides, leading to satisfactory performance of the LiRIDME technique on the neuroglobin sample. However, it should be noted that when using a high spin paramagnetic center as the fast‐relaxing species, as is the case for the triplet state, overtones of the dipolar frequencies are present. This makes the modulations in the dipolar trace corresponding to the dipolar frequency less clearly distinguishable as higher frequency overtone contributions are also present and distance analysis must take into account these overtone contributions.

In conclusion, in this work we demonstrate that an accurate determination of distance distributions can be achieved using the triplet state of ZnPP coupled to a nitroxide spin label in human neuroglobin. This is the first time that the feasibility of the dipolar experiment has been demonstrated for a paradigmatic protein belonging to the class of the hemeproteins, making clear use of the photoexcited triplet state. Our results have proven that LiRIDME can provide reliable information on the distance between nitroxides and triplet state chromophores in a similar fashion to LaserIMD. Both single‐frequency techniques become advantageous compared to LiDEER when the chromophore in the triplet state is characterized by short relaxation times.

Light induced PDS techniques should be seen as complementary to PDS techniques using stable radical spin centers. In particular, they are likely to be important for applications in spin systems which contain multiple spins as they enable a spin‐label to be turned on or switched off. In this way proof that it is possible to substitute the iron heme, which is spin‐active in its ground state, for the spin inactive ground state ZnPP in order to perform light‐induced PDS experiments is also a valuable result.

An important requisite to broaden the scope of the triplet spin labels in biological macromolecules is the availability of different light‐induced PDS techniques and the optimization of such pulse sequences, for example ReLaserIMD. The different techniques complement each other and, depending on the nature of the triplet spin label, can be used interchangeably, thereby taking advantage of specific properties of the stable radicals and triplet state present in a particular system, allowing the technique that yields the best performance to be used to characterize the biomolecule of interest.

## Experimental Section

The pulsed EPR measurements were carried out at Q‐band (34 GHz) on a Bruker ELEXSYS E580 spectrometer using a Bruker TII resonator. The experiments were performed at 20 K on glassy frozen solutions of ZnG19 (∼400 μM in deuterated Tris‐HCl buffer+66 % deuterated glycerol) and of the model peptide (∼100 μM in 98 % d‐methanol, 2 % D_2_O). All further experimental details are given in the Supporting Information.

## Conflict of interest

The authors declare no conflict of interest.

## Supporting information

As a service to our authors and readers, this journal provides supporting information supplied by the authors. Such materials are peer reviewed and may be re‐organized for online delivery, but are not copy‐edited or typeset. Technical support issues arising from supporting information (other than missing files) should be addressed to the authors.

Supplementary
