# Supplementary material for: Light‐Induced Pulsed EPR Dipolar Spectroscopy on a Paradigmatic Hemeprotein
Source: Chemphyschem. 2019 Mar 21;20(7):931–5. doi: 10.1002/cphc.201900139 (PMC6618045; doi:10.1002/cphc.201900139)
Supplement: Supplementary file 1 — Supplementary [file CPHC-20-931-s001.pdf]

Supporting Information

© Copyright Wiley-VCH Verlag GmbH & Co. KGaA, 69451 Weinheim, 2019

Light-Induced Pulsed EPR Dipolar Spectroscopy on a Paradigmatic Hemeprotein

Maria Giulia Dal Farra, Sabine Richert, Caterina Martin, Charles Larminie, Marina Gobbo, Elisabetta Bergantino, Christiane R. Timmel, Alice M. Bowen,* and Marilena Di Valentin*

Supporting Information for

Light-induced pulsed EPR dipolar spectroscopy on a paradigmatic Hemeprotein

Maria Giulia Dal Farra, Sabine Richert, Caterina Martin, Charles Larminie, Marina Gobbo, Elisabetta Bergantino, Christiane R. Timmel, Alice M. Bowen and Marilena Di Valentin.

Table of Contents

SAMPLE PREPARATION S2

MODEL PEPTIDE S2
HUMAN NEUROGLOBIN S2

EPR MEASUREMENTS AND DATA ANALYSIS S3

EPR MEASUREMENTS S3
EPR DATA ANALYSIS S4

NEUROGLOBIN SAMPLES CHARACTERIZATION S5

EPR DATA AND ANALYSES S5

ZNPP – MTSSL DISTANCE DISTRIBUTION IN ZNG19 ..... S5
LASERIMD PULSE SEQUENCE AND CORRESPONDING TIME TRACE S6
TIME-RESOLVED EPR AND PHOTOEXCITED FIELD-SWEPT ELECTRON SPIN ECHO S7
LIRIDME AND RELASERIMD DATA ON ZNG19 ..... S8
LIDEER ON ZNG19 ..... S8
PHASE MEMORY TIME S9
TRIPLET RELAXATION AND DECAY S9
NITROXIDE LONGITUDINAL RELAXATION IN ZNG19 ..... S10
OVERTONE ANALYSIS ON LIRIDME S10

SAMPLE PREPARATION

Model peptide

The model peptide was synthesized as described by Di Valentin *et al*.[1] The concentration of the EPR sample was 100 μM in 98% d-methanol, 2% D_2O. The EPR tube was sealed after several freeze-thaw cycles.

Human Neuroglobin

Rational design of the human neuroglobin mutant G19

Human neuroglobin contains three cysteine residues: Cys55 in position 5 of the D helix, Cys120 in position 19 of the G helix and Cys46 in position 7 of the CD loop, and all of them can be exploited as potential target for SDLS. To avoid multiple labeling, a mutant of neuroglobin, containing a single cysteine residue, has been expressed. The choice of the cysteine to label has been made on the basis of the X-ray crystallographic structure (PDB: 4MPM[2]) and of the available DEER data.[3] Cys46 was discarded because it is located in a random coil which is a highly flexible region and this could broaden the distance distribution between the two probes making the measurement of the dipolar trace more difficult. Cys55 was also eliminated because it is located in a buried position and this could diminish the labeling efficiency. As a further confirmation of these considerations, the two DEER traces recorded by M. Ezhevskaya *et al.*[3] labeling Cys55 in one mutant and Cys46 the other gave unsatisfactory signal-to-noise ratios and very poor modulation depths. For this reason, we expressed a neuroglobin mutant in *Escherichia coli*, were only Cys120 was present while Cys55 and Cys46 had been replaced by serine residues. Following the nomenclature by M. Ezhevskaya *et al.*[3] the mutant is called G19. After the expression of G19, the heme group was substituted with the Zn(II) zinc protoporphyrin (ZnPP) following the denaturation-purification-reconstitution procedure described by Scholler *et al.*[4] Subsequently the Cys120 was labeled with the nitroxide probe (1-oxyl-2,2,5,5-tetramethylpyrroline-3-methyl) methanethiosulfo-nate (MTSSL).

Expression, purification and preparation of the ZnPP-substituted mutant G19

The expression and purification protocol was performed following the procedure previously implemented.[4,5] The human neuroglobin DNA coding sequence cloned in the vector pET3a was kindly donated by Professor T. Burmester (Department of Biology, University of Hamburg). The GENEART®Site-Directed Mutagenesis System kit was used to make the G19 mutant: Cys46Ser/Cys55Ser. After the mutagenesis, the G19 coding sequence was cloned in pET28a, giving the plasmid pET28hNGB G19. The latter was finally used to transform the *Escherichia coli* strain BL21DE3 Codon Plus RP. The cells were grown at 25°C in TB medium containing 1.2% bactotryptone, 2.4% yeast extract, 0.4% glycerol, 72 mM potassium phosphate buffer, pH 7.5. 200 μg/ml ampicillin, 30 μg/ml chloramphenicol and 1 mM d-amino-levulinic acid were added to the medium. At OD_{600} = 0.8 the culture was induced by the isopropyl-1-thio-D- galactopyranoside to a final 0.4 mM concentration and cells were let growing overnight. The cells were then harvested, resuspended in lysis buffer (50 mM Tris-HCl pH 8.0, 1 mM EDTA, 0.5 mM dithiotreitol) and exposed to three freeze-thaw cycles. After that, sonication was done until the cells completely lysed. The extract was clarified by low (10 min at 10,000 g) and high (60 min at 105,000 g) speed centrifugation. The obtained supernatant was collected and fractionated by 60% ammonium sulphate precipitation. The pellets were dialyzed against 5 mM Tris-HCl pH 8.5 and loaded onto a DEAE Sepharose fast flow column for ion exchange chromatography. The recombinant neuroglobin G19 was eluted by 200 mM NaCl from the Sepharose column, concentrated by Amicon filtration (PM10) and passed through a Sephacryl S200 column for gel filtration. SDS-PAGE was done to test the protein purity and the Bradford reagent was used for measuring protein yield. The ZnPP-substitution was performed following the procedure described by Scholler *et al.*[4] ZnPP was purchased from Sigma Aldrich. All manipulations were performed in the dark because of the light sensitivity of ZnPP. In a first step, the original heme group was removed. In order to prepare the apo-G19, a cold acid acetone solution (50 mL of acetone + 40 μL of HCl 6N) was added drop by drop to G19, constantly stirred at –20°C. White apo-protein flakes were centrifuged for 1 min at 1200 g to separate them from the red heme solution. The supernatant was discarded. The apo-protein was washed with cold acid acetone and again centrifuged. The sediment was dissolved in a minimum amount of water and dialyzed 24 h in $NaHCO_3$ 1 mM, DTT 0.1 mM and overnight in buffer 5 mM Tris-HCl pH 8.5. The obtained apo-protein was used to prepare ZnPP-substituted neuroglobin. ZnPP was solubilized in 0.1 M NaOH and dissolved in buffer 5 mM Tris-HCl pH 8.5. This was added to the apo-neuroglobin solution, which was then incubated for 4 hours, dialyzed overnight against 5 mM Tris-HCl buffer at pH 8.5 and concentrated

by Amicon filtration (PM10). The yield of ZnPP-substituted neuroglobin from apo-protein resulted to be 50% (measured by ZnPP absorbance using ε_{424}= 122 mM^{-1} cm^{-1}). The residual apo-protein, remaining denaturated or mis-folded, was easily eliminated by centrifugation.

Spin labeling

Before starting the spin labeling procedure, the protein was incubated for 30 minutes in a 10 mM DTT solution in 5 mM Tris-HCl pH 8.5 buffer. The sample buffer was exchanged with 5 mM Tris-HCl pH 8.5 using a desalting column. Spin labeling was carried out by adding a 10-fold molar excess of MTSL to the single cysteine G19 mutant. The sample was incubated first for 2 hours at room temperature and then overnight at 4°C. It was then washed in a desalting column to remove the unbound spin label. The labeling yield, determined by CW-EPR, was 68%. The buffer for the EPR sample was prepared with deuterated water (Cambridge Isotopes) and oxygen was removed with the glucose/glucose oxidase/catalase enzymes. 66% v/v of deuterated glycerol (Sigma Aldrich), previously degassed via freeze-thaw cycles, was added to the solution to obtain a transparent matrix. The final sample concentration was 400 μM.

EPR MEASUREMENTS AND DATA ANALYSIS

EPR measurements

The pulsed and time-resolved EPR experiments were conducted on a Bruker ELEXSYS E580 spectrometer fitted with a Spinjet Arbitrary Waveform Generator (AWG) using a TII resonator at Q-band (34 GHz). The temperature was maintained at 20 K using liquid helium and a CF935 cryostat with an Oxford Instruments ITC103 temperature controller. Laser excitation of the samples was performed using an OPO pumped by the third harmonic of a Nd:YAG laser (Opotek, Opolette355), operated at a repetition rate of 20 Hz (5 ns pulses). Laser pulse energies of 2 mJ were used at an excitation wavelength of 512 nm for the model peptide and 552 nm for ZnG19.

Time-resolved EPR experiments were collected as a bi-dimensional dataset (magnetic field vs time) in transient mode of the Bruker ELEXSYS E580 spectrometer using a microwave power of 0.1002 mW (23 dB attenuation) and signals were recorded for a total time window of 1.3 ms to monitor the time evolution. The data presented in Figure S4 is the average of 1000 ns of the time evolution around the signal maximum in the time domain which occurs immediately after the laser pulse.

For the field swept electron spin-echo experiments a standard Hahn echo sequence (laser pulse - DAF - π/2 - τ - π - τ - *echo*) was employed for both ZnG19 and the model peptide, with a nominal length of 28 ns for all microwave pulses and a τ value of 200 ns. The delay after the laser flash (DAF) was set to 1600 ns. The data were collected with 5 scans and 50 shots-per-point. The phase memory time of the nitroxide and of the triplet state of the porphyrin derivatives were measured, on both samples, with a 2-pulse echo decay sequence (π/2 – (τ + dt) - π - (τ + dt) - *echo*), adding the laser photoexcitation at the beginning of the pulse sequence (DAF = 1600 ns), with 1 shot-per-point and 5 scans. The time increment and the inter-pulse delay were 8 ns and 200 ns, respectively. A two-step phase cycle was employed to remove receiver offsets. The relaxation and decay of the ZnPP triplet state in ZnG19 were measured at the field positions corresponding to the zero-field splitting canonical orientations, integrating the Hahn echo at increasing values of the DAF. The data were measured accumulating 50 scans with 1 shot-per-point. A time increment of 60 μs was used along both the X^- and Y^- zero-field splitting canonical transitions, while an increment of 5 μs was used for the Z^+ transition.

The LaserIMD experiment on the model peptide was performed with the pulse sequence proposed by Hintze *et al.*[6] (π/2 - τ - π - t - laser pulse - (τ – t) - *echo*) with a τ of 1500 ns and 28 ns microwave pulse lengths. For the ReLaserIMD experiments a pulse scheme: π/2 - τ_1 - π - t - laser pulse - (τ_1 + τ_2 – t) - π - τ_2 - *echo* was used for both samples, with 28 ns microwave pulse lengths, a τ_1 of 1500 ns and a τ_2 200 ns. In both cases a two-step phase cycle was applied to remove receiver offsets. The LaserIMD and the ReLaserIMD traces of the model peptide were accumulated for ~2 hours while the ReLaserIMD trace of ZnG19 was accumulated for ~13 hours. In both the experiments the working field was set at the maximum of the nitroxide spectrum. For the LiDEER experiment on ZnG19 a four-pulse sequence coupled to laser photoexcitation was applied (laser pulse - DAF - π /2 - τ_1 - π - t - π_{pump} - (τ_1 + τ_2 – t) - π - τ_2 - *echo*). The corresponding parameters are: DAF = 1600 ns, microwave pulse lengths = 28 ns, τ_1 = 200 ns and a τ_2 = 1300 ns. The difference between the pump (nitroxide maximum) and observer (porphyrin triplet state, at the Y^- zero-field splitting canonical

transition) frequency was set to 440 MHz A two-step phase cycle was applied to remove receiver offsets while deuterium nuclear modulations were suppressed using an 8 step τ_1 cycle with 16 ns increment steps. The total measuring time was ~20 hours. The LiRIDME experiment on ZnG19 was performed with the five-pulse sequence coupled to laser photoexcitation (laser pulse - DAF - π /2 - τ - π - τ - t - π /2 - T - π/2 - (τ – t) - π - τ – *echo*), setting the field at the maximum of the nitroxide spectrum. The corresponding parameters are: DAF = 1600 ns, microwave pulse lengths = 28 ns, τ = 200 ns, T = 80 ns. In order to eliminate the contribution of unwanted echoes, an eight-step phase cycle was applied. The measuring time was about ~20 hours. In all the pulsed dipolar experiments, the time increment of the traces was 8 ns.

EPR data analysis

LaserIMD, ReLaserIMD time traces on the model peptide were analyzed using the DeerAnalysis2018 routine.[7] The study on the determination of the zero-time in the LaserIMD and the ReLaserIMD time traces, was carried out selecting different zero times, for both the datasets, and repeating the procedure of background fitting and Tikhonov regularization. For the LaserIMD, nine different the zero times, separated by 4 ns, were picked around the change of slope of the curve. For the ReLaserIMD, three different zero times, separated by 4 ns, were selected around the symmetry axis of the first modulation. The primary data from the experiments were background corrected by fitting an exponential decay function to the data. In both cases, the portion of the trace, employed for the background fitting, was kept constant for all the trials. The regularization parameters were determined with the GCV criterion.[8]

LiDEER, ReLaserIMD time traces on ZnG19 were analyzed using the DeerAnalysis2018 routine,[7] while LiRIDME data were analyzed in OvertoneAnalysis.[9] The traces were elaborated dividing, from raw data, the background contribution and processing the form factor by Tikhonov regularization to extract the distance distributions. A third-degree polynomial function was employed to effectively remove the background contribution, from the ReLaserIMD, while exponential functions, with dimensions d = 3 and d = 6.8, were used for the LiDEER and the LiRIDME data, respectively. The distance analysis was performed, in the case of the ReLaserIMD and the LiDEER data, by Tikhonov regularization, using the GCV criterion for determining the optimum regularization parameter. The Tikhonov regularization implemented in OvertoneAnalysis was employed to analyze the LiRIDME form factor. The values of the first and the second harmonic coefficients, were selected repeating the fitting of the LiRIDME form factor for different values of the coefficients and selecting those giving the best result in term of suppression of artifacts in the distance distribution and fit of the trace (see Figure S6). The value of the regularization parameter was selected looking for the best compromise between the goodness of the form factor fitting and smoothness of the distance distribution.

The fits of the relaxation data ware performed in MATLAB.

NEUROGLOBIN SAMPLES CHARACTERIZATION

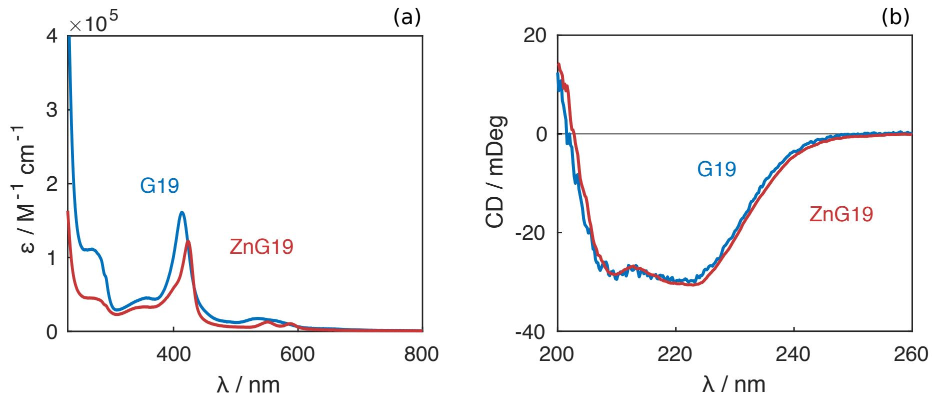

Figure S1: UV-Vis (a) and circular dichroism (b) spectra of of G19 (blue) and ZnG19 (red) 10 μM in Tris-HCl buffer.

EPR DATA AND ANALYSES

ZnPP – MTSSL distance distribution in ZnG19

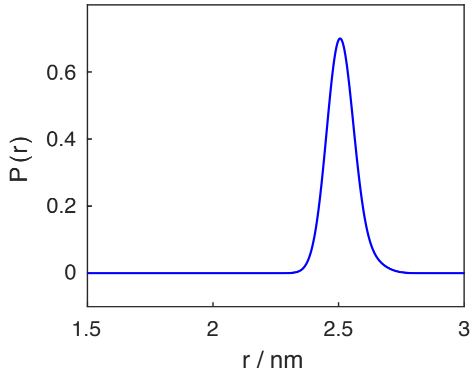

Figure S2. Distance distribution predicted by MMM,[10] using the pdb structure 4MPM,[2] between the MTSSL label, attached to Cys120, assuming a temperature of 175 K, and the center of the ZnPP.

LaserIMD pulse sequence and corresponding time trace

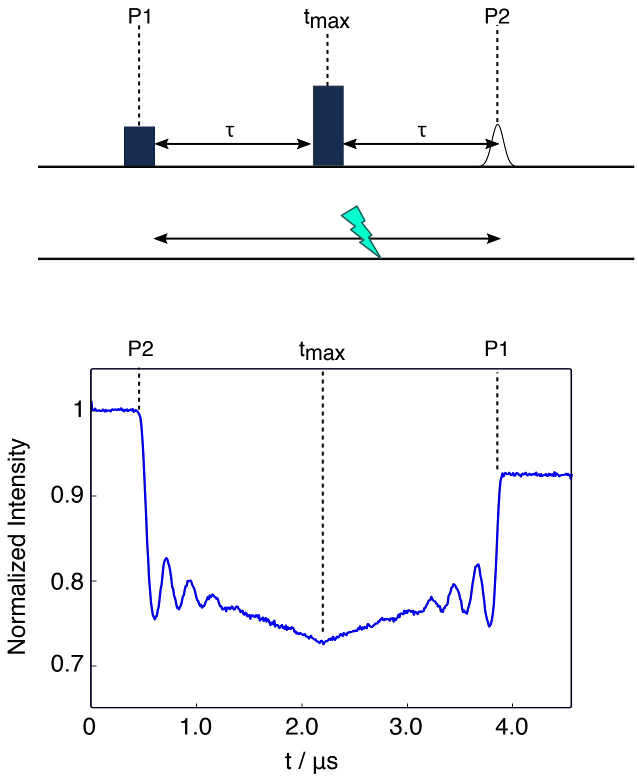

Figure S3. LaserIMD pulse scheme and corresponding dipolar time trace measured on the model peptide.

Time-resolved EPR and photoexcited field-swept electron spin echo

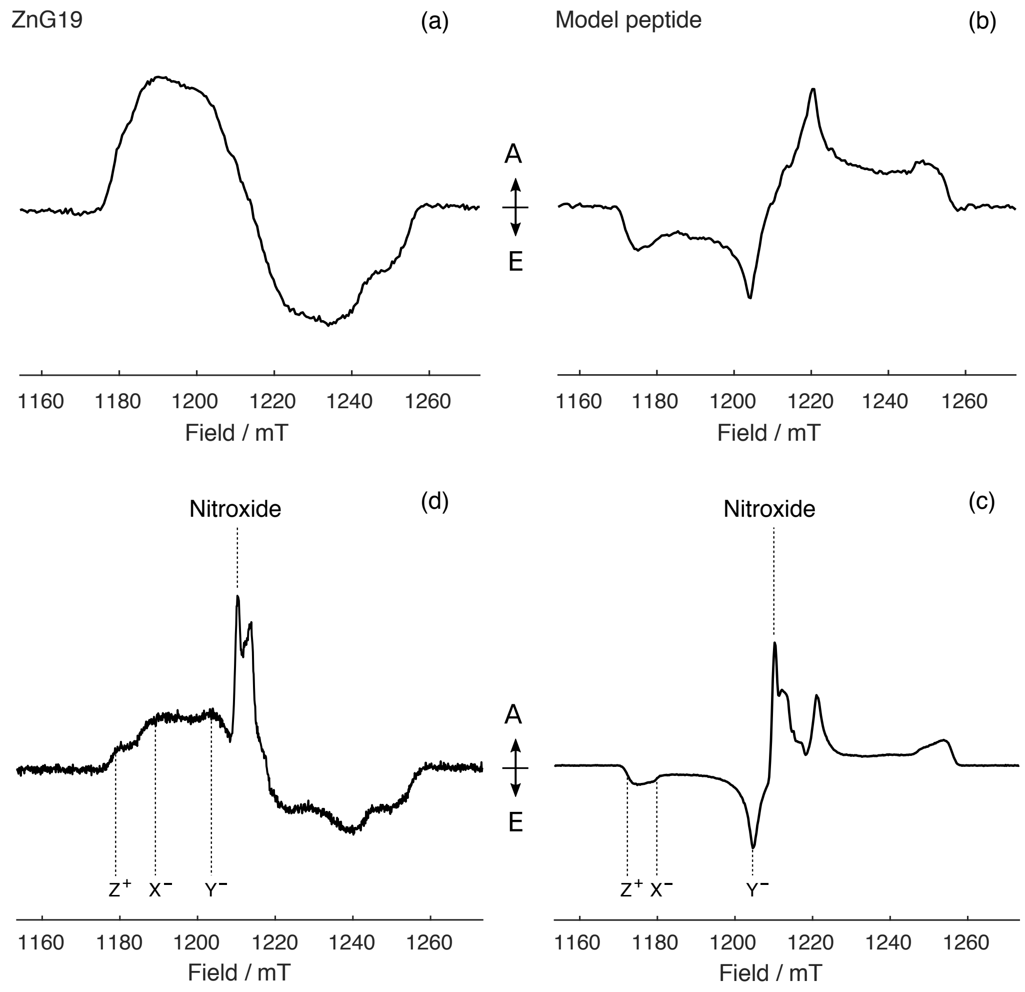

Figure S4. Top panel: Time-resolved EPR spectra for ZnG19 (a) and the model peptide (b). For ZnG19, the zero-field splitting parameters of the ZnPP triplet state (|D| = 1078 MHz, |E| = 181 MHz) and the spin polarization AAAEEE are in agreement with those reported for the ZnPP triplet state by M. Fahnenschmidt *et al.*[11] For the model peptide, the zero-field parameters of the tetraphenylporphyrin triplet state (|D| = 1183 MHz, |E| = 241 MHz) and the spin polarization pattern EAEAEA are the same as reported previously.[1] Bottom panel: photoexcited field-swept electron spin echo spectra for ZnG19 (d) and the model peptide (c). The spectra, recorded optimizing the microwave pulse power on the nitroxide radical, show the broad contribution from the porphyrin triplet state and the narrow signal at the center due to the nitroxide radical. The low field triplet canonical transitions are labeled in all the spectra based on D>0, E<0. The "+" and "–" signs refer to the triplet transitions that take place between the levels $|T_0\rangle$ - $|T_{+1}\rangle$ and $|T_0\rangle$ - $|T_{-1}\rangle$, respectively.

LiRIDME and ReLaserIMD data on ZnG19

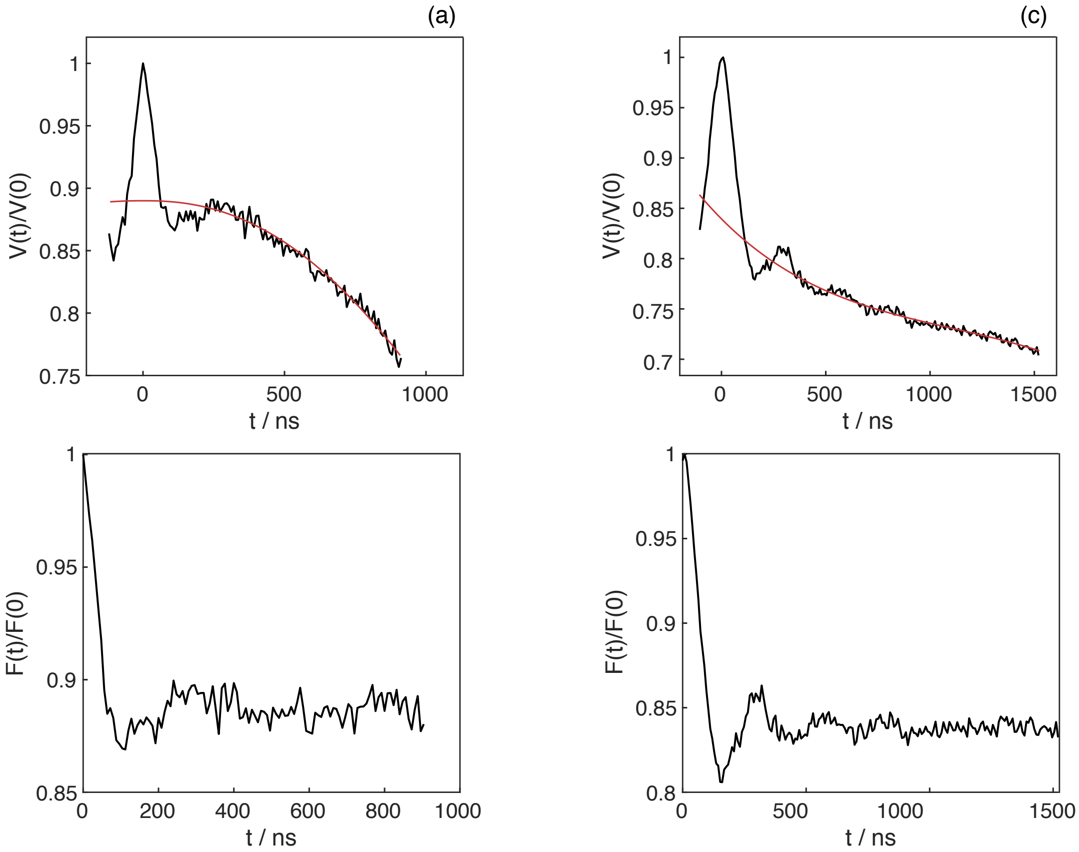

Figure S5: LiRIDME (a) and ReLaserIMD (b) data recorded on ZnG19 under the experimental conditions described in EPR measurements and data analyses. For both datasets the raw data (black) with the corresponding background fit are displayed in the top panel, while the form factors obtained after background division are shown in the bottom panel.

LiDEER on ZnG19

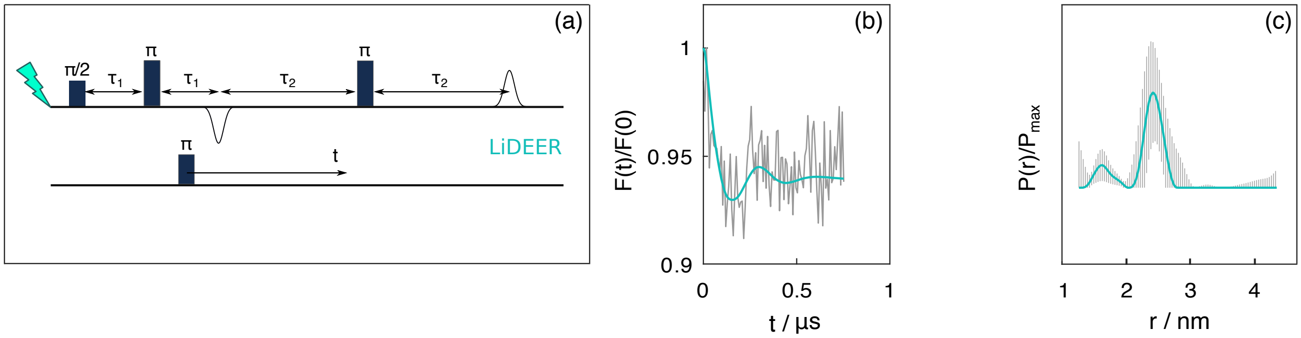

Figure S6. LiDEER data measured on ZnG19: (a) pulse schemes, (b) form factor (grey) and best fit (turquoise), (c) corresponding distance distribution. The distance analysis has been performed with DeerAnalysis. The error in the distance distribution has been obtained using the validation procedure by varying the starting point for the background fitting between 300 and 500 ns and adding 50% of the original noise.

Phase memory time

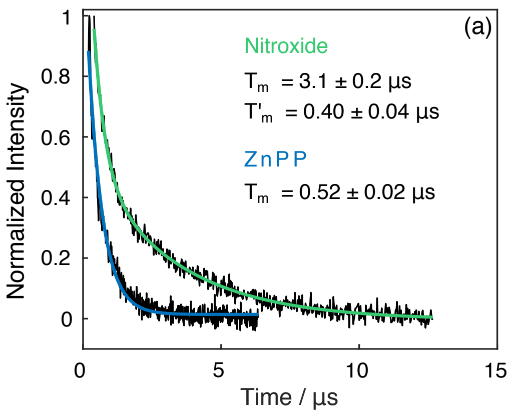

Figure S7. (a) 2-pulse electron spin echo decay data, recorded under photoexcitation, and corresponding fits recorded at the maximum of the nitroxide spectrum (green) and along the Y^- canonical positions of the porphyrin triplet state (blue). A mono-exponential decay function was used to fit the triplet phase memory time, while a bi-exponential decay was used for the nitroxide. (b) 2-pulse electron echo decay data, displayed with the corresponding fits, recorded, under photoexcitation, along the Y^- canonical positions of the tetraphenylporphyrin (TPP) triplet state in the model peptide (red) and of the ZnPP triplet state in ZnG19 (blue). A mono-exponential decay function was used to fit the triplet phase memory time.

Triplet relaxation and decay

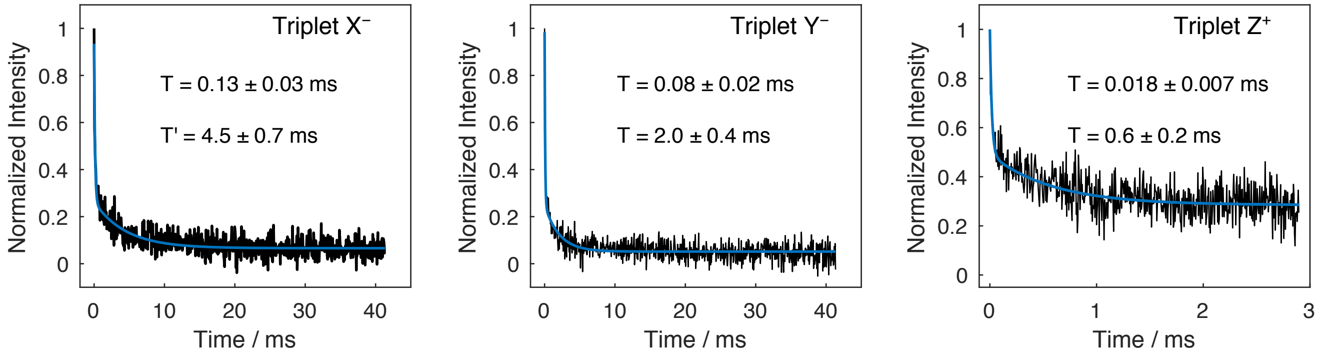

Figure S8. Integrated Hahn-echo intensity as a function of the delay after the laser pulse of the triplet state of ZnPP in ZnG19. The traces have been recorded at the field positions corresponding to the triplet state canonical orientations of ZnPP indicated in Figure S1. The data have been fitted with a bi-exponential decay function.

Nitroxide longitudinal relaxation in ZnG19

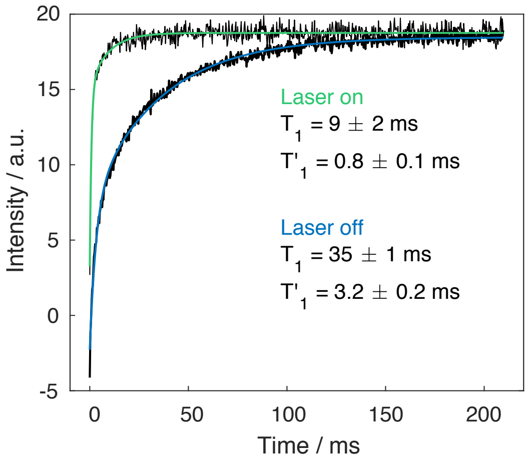

Figure S9. Inversion recovery data and corresponding fits, recorded at maximum of the nitroxide spectrum in ZnG19, both in the presence (green) and in the absence (blue) of laser photoexcitation. A bi-exponential decay function was used to fit the data.

Overtone analysis on LiRIDME

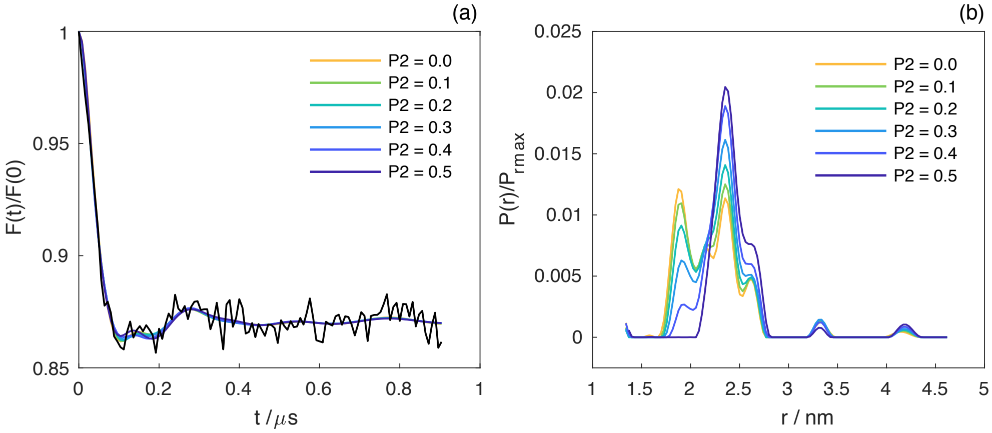

Figure S10. LiRIDME on ZnG19 and distance analysis, performed with OvertoneAnalysis by using different second harmonic coefficients, P2. The value of the first harmonic coefficient is P1 = 1 – P2. (a) Form factor and best fits calculated for different values of the harmonic coefficients and (b) corresponding distance distributions.

REFERENCES

[1] M. Di Valentin, M. Albertini, E. Zurlo, M. Gobbo, D. Carbonera, *J. Am. Chem. Soc.* **2014**, *136*, 6582–6585.

[2] B. G. Guimarães, D. Hamdane, C. Lechauve, M. C. Marden, B. Golinelli-Pimpaneau, *Acta Crystallogr. Sect. D Biol. Crystallogr.* **2014**, *70*, 1005–1014.

[3] M. Ezhevskaya, E. Bordignon, Y. Polyhach, L. Moens, S. Dewilde, G. Jeschke, S. Van Doorslaer, *Mol. Phys.* **2013**, *111*, 2855–2864.

[4] D. M. Scholler, M.-Y. R. Wang, B. M. Hoffman, *Methods Enzymol.* **1978**, *52*, 487–493.

[5] S. Dewilde, K. Mees, L. Kiger, C. Lechauve, M. C. Marden, A. Pesce, M. Bolognesi, L. Moens, *Methods Enzymol.* **2008**, *436*, 341–357.

[6] C. Hintze, D. Bücker, S. Domingo Köhler, G. Jeschke, M. Drescher, *J. Phys. Chem. Lett.* **2016**, *7*, 2204–2209.

[7] G. Jeschke, V. Chechik, P. Ionita, A. Godt, H. Zimmermann, J. Banham, C. R. Timmel, D. Hilger, H. Jung, *Appl. Magn. Reson.* **2006**, *30*, 473–498.

[8] T. H. Edwards, S. Stoll, *J. Magn. Reson.* **2018**, *288*, 58–68.

[9] K. Keller, V. Mertens, M. Qi, A. I. Nalepa, A. Godt, A. Savitsky, G. Jeschke, M. Yulikov, *Phys. Chem. Chem. Phys.* **2017**, *19*, 17856–17876.

[10] Y. Polyhach, E. Bordignon, G. Jeschke, *Phys. Chem. Chem. Phys.* **2011**, *13*, 2356–2366.

[11] M. Fahnenschmidt, R. Bittl, R. Bittl, E. Schlodder, W. Haehnel, W. Lubitz, *Phys. Chem. Chem. Phys.* **2001**, *3*, 4082–4090.
